# Disruption of IL-21 Signaling Affects T Cell-B Cell Interactions and Abrogates Protective Humoral Immunity to Malaria

**DOI:** 10.1371/journal.ppat.1004715

**Published:** 2015-03-12

**Authors:** Damián Pérez-Mazliah, Dorothy Hui Lin Ng, Ana Paula Freitas do Rosário, Sarah McLaughlin, Béatris Mastelic-Gavillet, Jan Sodenkamp, Garikai Kushinga, Jean Langhorne

**Affiliations:** Division of Parasitology, MRC National Institute for Medical Research (NIMR), London, United Kingdom; Case Western Reserve University, UNITED STATES

## Abstract

Interleukin-21 signaling is important for germinal center B-cell responses, isotype switching and generation of memory B cells. However, a role for IL-21 in antibody-mediated protection against pathogens has not been demonstrated. Here we show that IL-21 is produced by T follicular helper cells and co-expressed with IFN-γ during an erythrocytic-stage malaria infection of *Plasmodium chabaudi* in mice. Mice deficient either in IL-21 or the IL-21 receptor fail to resolve the chronic phase of *P*. *chabaudi* infection and *P*. *yoelii* infection resulting in sustained high parasitemias, and are not immune to re-infection. This is associated with abrogated *P*. *chabaudi*-specific IgG responses, including memory B cells. Mixed bone marrow chimeric mice, with T cells carrying a targeted disruption of the *Il21* gene, or B cells with a targeted disruption of the *Il21r* gene, demonstrate that IL-21 from T cells signaling through the IL-21 receptor on B cells is necessary to control chronic *P*. *chabaudi* infection. Our data uncover a mechanism by which CD4+ T cells and B cells control parasitemia during chronic erythrocytic-stage malaria through a single gene, *Il21*, and demonstrate the importance of this cytokine in the control of pathogens by humoral immune responses. These data are highly pertinent for designing malaria vaccines requiring long-lasting protective B-cell responses.

## Author Summary

The importance of antibody and B-cell responses for control of the erythrocytic-stage of the malaria parasite, *Plasmodium*, was first described when immune serum, passively transferred into *Plasmodium falciparum*-infected children, reduced parasitemia. This was later confirmed in experimental models in which mice deficient in B cells were unable to eliminate erythrocytic-stage infections. The signals required to activate these protective long-lasting B cell responses towards *Plasmodium* have not been investigated. IL-21 has been shown to be important for development of B-cell responses after immunization; however, a direct requirement for IL-21 in the control of infection via B-cell dependent mechanisms has never been demonstrated. In this paper, we have used mouse models of erythrocytic *P*. *chabaudi* and *P*. *yoelii* 17X(NL) infections in combination with IL-21/IL-21R deficiency to show that IL-21 from CD4^+^ T cells is required to eliminate *Plasmodium* infection by activating protective, long-lasting B-cell responses. Disruption of IL-21 signaling in B cells prevents the elimination of the parasite resulting in sustained high parasitemias, with no development of memory B-cells, lack of antigen-specific plasma cells and antibodies, and thus no protective immunity against a second challenge infection. Our data demonstrate the absolute requirement of IL-21 for B-cell control of this systemic infection. This has important implications for the design of vaccines against *Plasmodium*.

## Introduction

Malaria is the leading parasitic cause of morbidity and mortality worldwide; about half of the world's population is at risk of infection [1]. There is an urgent need for an effective vaccine able to bring about high levels of protection.

Immunity to the erythrocytic stages of malaria is thought to be primarily dependent on an antibody response. In endemic areas of *Plasmodium falciparum* transmission, there are associations between *Plasmodium*-specific antibody responses and protection against infection [2–5]. Elimination of the erythrocytic-stages of *Plasmodium falciparum* in infected children can be achieved by passive transfer of immune serum [2, 6], and studies in experimental models show that B cells and antibodies are important for elimination of chronic infections, and immunity to re-infection [7, 8]. A better understanding of the signals underlying activation of protective, long lasting, B-cell responses would be of great value in malaria vaccine development.

The cytokine IL-21, produced by follicular helper CD4^+^ T cells (Tfh) and other cells, is important for the generation of B-cell responses in germinal centers (GC), isotype switching, affinity maturation, antibody production, and development of memory B cells (MBC) [9, 10]. However, a requirement of IL-21 for activation and maintenance of Tfh cell is still controversial [11–23]. Most of our knowledge about the role of IL-21 in humoral responses has come from studies using immunization with protein antigens, where IL-21 is critical for the development of a T-cell dependent IgG response in GCs [11, 15, 16, 21, 23, 24]. Contrary to its importance in generating B cell responses after immunization, IL-21 seems not to be necessary for all aspects of T-cell-dependent B cell responses in different infection models [14, 19, 20, 22, 25, 26].

An investigation into Tfh cell development and the role of IL-21 in malaria has not been carried out, but this would be an excellent infection model in which to determine the importance of IL-21 in protective humoral immunity to a systemic pathogen, and would shed light on the induction, control and impairment of humoral responses in malaria. Here we have used a mouse model of malaria, *Plasmodium chabaudi chabaudi AS* in C57BL/6 mice, and have shown that IL-21 and Tfh cells are prominently induced and maintained in an erythrocytic-stage infection, suggesting that this crucial element of the humoral response is not impaired. Tfh cells producing IL-21 are multifunctional, with a majority also producing IFN-γ. Importantly, IL-21 produced by CD4^+^ T cells, acting directly on B cells, is crucial for triggering protective long-lasting *Plasmodium*-specific IgG B cell responses that are required to control and resolve the chronic phase of erythrocytic-stage malaria infection and for immunity to re-infection.

## Results

### IL-21 signaling is essential to control the chronic phase of blood stage *P*. *chabaudi* infection

Injection of 10^5^ red blood cells (rbc) infected with *P*. *chabaudi* (irbc) into C57BL/6 mice gives rise to an erythrocytic infection, with an early acute parasitemia occurring at day 8 post-infection. Thereafter, the infection is rapidly controlled, reaching very low parasitemia levels by day 20 post-infection. This is followed by a chronic phase of infection, characterized by a prolonged sub-patent parasitemia with small patent recrudescence for up to 60 days before parasite elimination [27].

**Fig 1:**
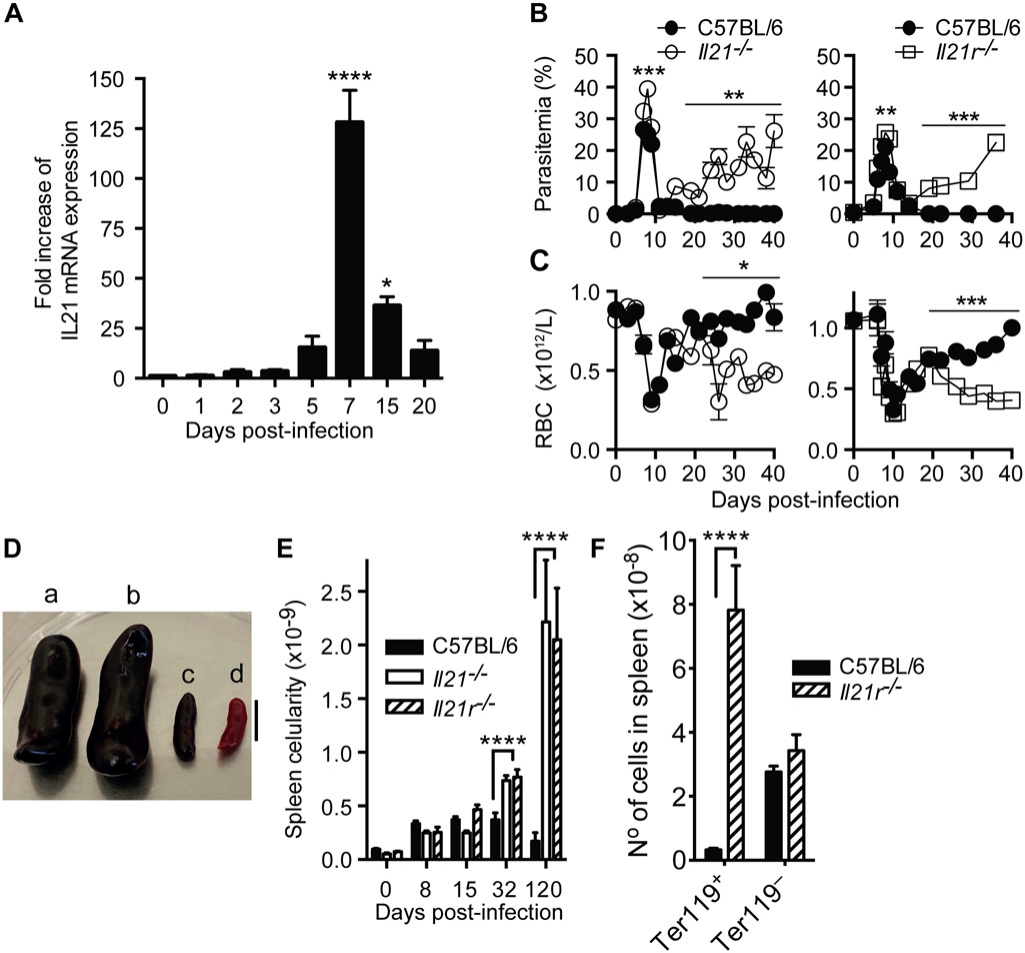
IL-21 is produced during *P*. *chabaudi* infection and required to control chronic infection. (A) IL-21 mRNA in spleen cells of *P*. *chabaudi*-infected mice measured by real-time quantitative RT-PCR. Parasitemia (B) and total rbc counts (C) were determined in WT C57BL/6 (closed circles), *Il21* ^*-/-*^ (open circles) and *Il21r* ^*-/-*^ (open squares) mice. (D) Individual examples of spleens from *Il21r* ^*-/-*^ (a) *Il21* ^*-/-*^ (b) and WT C57BL/6 (c) mice at day 120 post-infection, and a spleen from an age-matched WT C57BL/6 naïve mouse (d). Bar, 1 cm. (E) Total number of nucleated live splenocytes were determined with a hemocytometer in WT C57BL/6 (black bars), *Il21* ^*-/-*^ (open bars) and *Il21r* ^*-/-*^ (stripped bars) mice. (F) Numbers of Ter119^+^ and Ter119^–^ cells in the spleen of WT C57BL/6 (black bars) and *Il21r* ^*-/-*^ (striped bars) at day 32 post-infection. Data are representative of two or more independent experiments and are obtained in groups of 5–10 mice per time point. Statistical significance was obtained using Mann Whitney U test or Kruskal-Wallis test. *, P<0.05; **, P<0.01; ***, P<0.001; ****, P<0.0001. Error bars correspond to mean ± SEM.

To explore a role for IL-21 during erythrocytic-stage *P*. *chabaudi* infection, we first determined the pattern of IL-21 mRNA expression in spleens of C57BL/6 mice during a *P*. *chabaudi* infection by real-time quantitative RT-PCR. Although IL-21 mRNA was detected over basal naïve levels as early as 2 days post-*P*. *chabaudi* infection, there was a striking increase in the spleen by day 7 post-infection, when IL-21 mRNA levels were approximately 130-fold higher than the basal level. IL-21 mRNA decreased thereafter, but remained higher than the basal level for at least 20 days (Fig 1 A).

We next investigated whether IL-21, or signaling through its receptor, was necessary to control a primary *P*. *chabaudi* infection. C57BL/6 mice carrying a targeted deletion of the *Il21* gene, or its receptor, *Il21r*, were infected as described above. They were able to control the acute phase of *P*. *chabaudi* infection, despite showing slightly but significantly higher peak parasitemias than infected WT C57BL/6 mice (Fig 1 B). Similar to WT C57BL/6 mice, both *Il21* ^*-/-*^ and *Il21r* ^*-/-*^ mice had very low parasitemias by 11–14 days post-infection. However, in stark contrast to WT mice, they developed sustained high parasitemias during the chronic phase of infection (e.g., parasitemias of 17% in *Il21* ^*-/-*^ and 23% in *Il21r* ^*-/-*^ mice compared with less than 0.04% in WT C57BL/6 at day 36 post-infection) (Fig 1 B). These high parasitemias were maintained in both *Il21* ^*-/-*^ and *Il21r* ^*-/-*^ after 120–150 days of infection (44±4% in *Il21* ^*-/-*^ and 56±3% in *Il21r* ^*-/-*^ mice at day 120 post-infection), without mortality. The non-resolving *P*. *chabaudi* infection was accompanied by increased anemia in both *Il21* ^*-/-*^ and *Il21r* ^*-/-*^ mice from days 19–22 post-infection onwards (Fig 1 C).

*P*. *yoelii* 17X(NL) gives rise to a non-lethal erythrocytic infection in WT C57BL/6 mice that is completely cleared after the acute phase without showing a chronic phase (S1 Fig, A and B). Similar to the *P*. *chabaudi* infection, both *Il21* ^*-/-*^ and *Il21r* ^*-/-*^ mice failed to clear a *P*. *yoelii* 17X(NL) erythrocytic infection, and developed sustained high parasitemias (S1 Fig, A and B). These data show that the requirement for IL-21 signaling is necessary to control infection with different *Plasmodium* species.

*P*. *chabaudi*-infected *Il21* ^*-/-*^ and *Il21r* ^*-/-*^ mice showed significantly greater spleen cellularity compared with WT C57BL/6 controls as early as 32 days post-infection, and dramatic splenomegaly at later time points (Fig 1, D and E). Despite increased anemia and splenomegaly, neither *Il21* ^*-/-*^ nor *Il21r* ^*-/-*^ mice had any other clinical signs. At day 32 post-infection, the numbers of Ter119^+^ erythrocytic cells in the spleen in the absence of IL-21 signaling were dramatically increased (Fig 1F), thus contributing to the large splenomegaly observed in *Il21* ^*-/-*^ and *Il21r* ^*-/-*^ mice from day 32 post-infection onwards.

In addition, at this time the numbers of NK cells, granulocytes and monocytes were greater in the *Il21r* ^*-/-*^ spleens compared with WT C57BL/6 controls (S2 Fig). On the other hand, the numbers of B cells in the spleen from *Il21r* ^*-/-*^ mice were substantially reduced compared to WT C57BL/6 control (S2 Fig).

Taken together, these data demonstrate a central role for IL-21 signaling in the resolution of erythrocytic-stage *P*. *chabaudi* and *P*. *yoelii* 17X(NL) infections.

### IL-21 is produced by CD4^+^ T cells during *P*. *chabaudi* infection, and co-expressed with IFN-γ and IL-10

**Fig 2:**
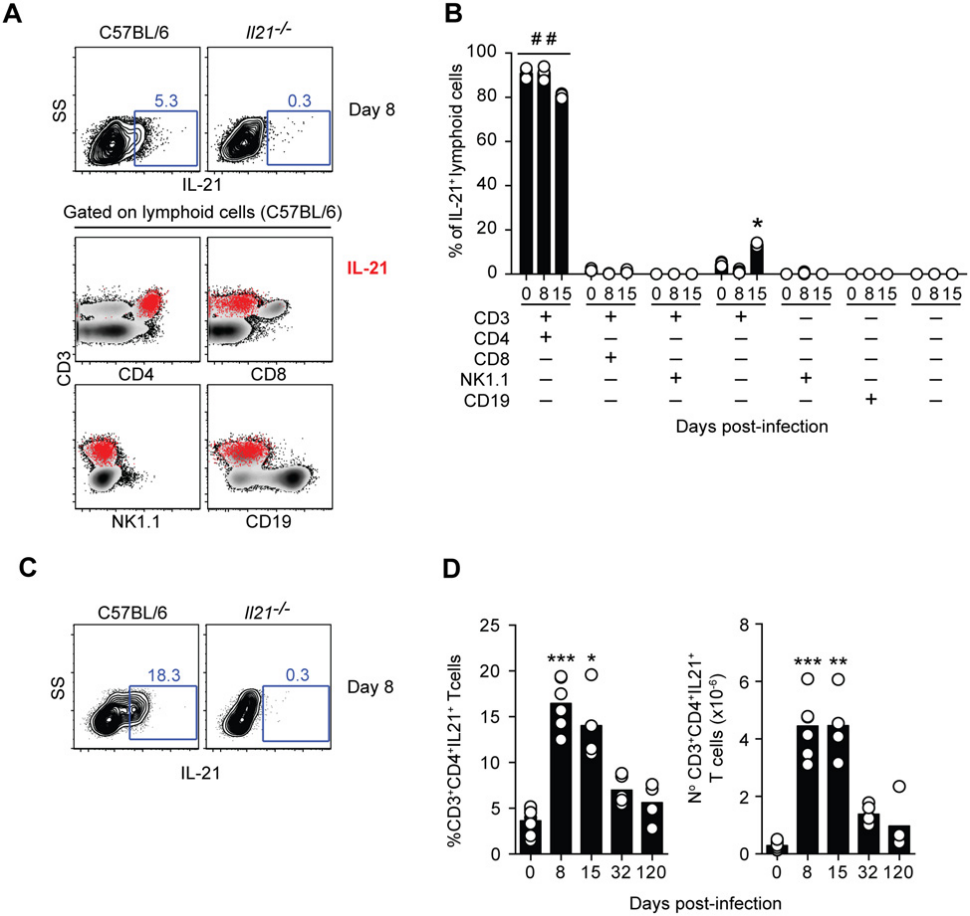
IL-21 is produced by CD4^+^ T cells during *P*. *chabaudi* infection. (A) Flow cytometry plots showing individual examples of IL-21 expression on mononuclear cells from WT C57BL/6 and *Il21* ^*-/-*^ mice at day 8 post-infection (top row). For the gating strategy, singlet cells were first selected, followed by live cells and mononuclear cells. In the bottom row, the IL-21-producing mononuclear cells detected in WT C57BL/6 mice, identified by red dots, were overlaid on the plots corresponding to the different combinations of surface biomarkers. (B) Cumulative data showing the differential combination of expression (+) or absence of expression (–) of each surface marker (indicated in the bottom left) on IL-21-producing mononuclear cells. (C) Flow cytometry plots showing individual examples of IL-21 expression on CD3^+^CD4^+^ T cells at day 8 post-infection. (D) Cumulative data showing the percentage (left) and total numbers (right) of IL-21-producing CD4^+^ T cells in the spleen of WT C57BL/6 mice at different days post-infection. Data are representative of at least two independent experiments and were obtained in groups of 4–5 mice per time point. Statistical significance was obtained using the Kruskal-Wallis test comparing each time point with its respective basal level (day 0 post-infection) (*, P<0.05; **, P<0.01; ***, P<0.001); or comparing each surface marker combination with every other surface marker combination within each time point (# #, P<0.01). Bars represent median values.

To identify which cells were responsible for the production of IL-21, intracellular cytokine staining and multiparameter flow cytometry performed on splenic cells from WT C57BL/6 mice revealed that IL-21 production was observed only in CD4^+^ T cells throughout the acute phase of a *P*. *chabaudi* infection (Fig 2, A and B). At no point during the infection was IL-21 detectable in NK cells, CD19^+^ cells or CD3^–^NK1.1^–^ cells. In accordance with the kinetics observed for IL-21 mRNA expression (Fig 1 A), the percentage and total number of IL-21-producing CD4^+^ T cells in the spleen increased early after infection, (Fig 2, C and D). Both percentages and total numbers decreased thereafter, but remained higher than basal naïve levels at least up to day 32 post-infection. By 120 days post-infection, the frequency and total numbers of IL-21-producing CD4^+^ T cells in the spleen were comparable to those observed in naïve WT C57BL/6 mice.

**Fig 3:**
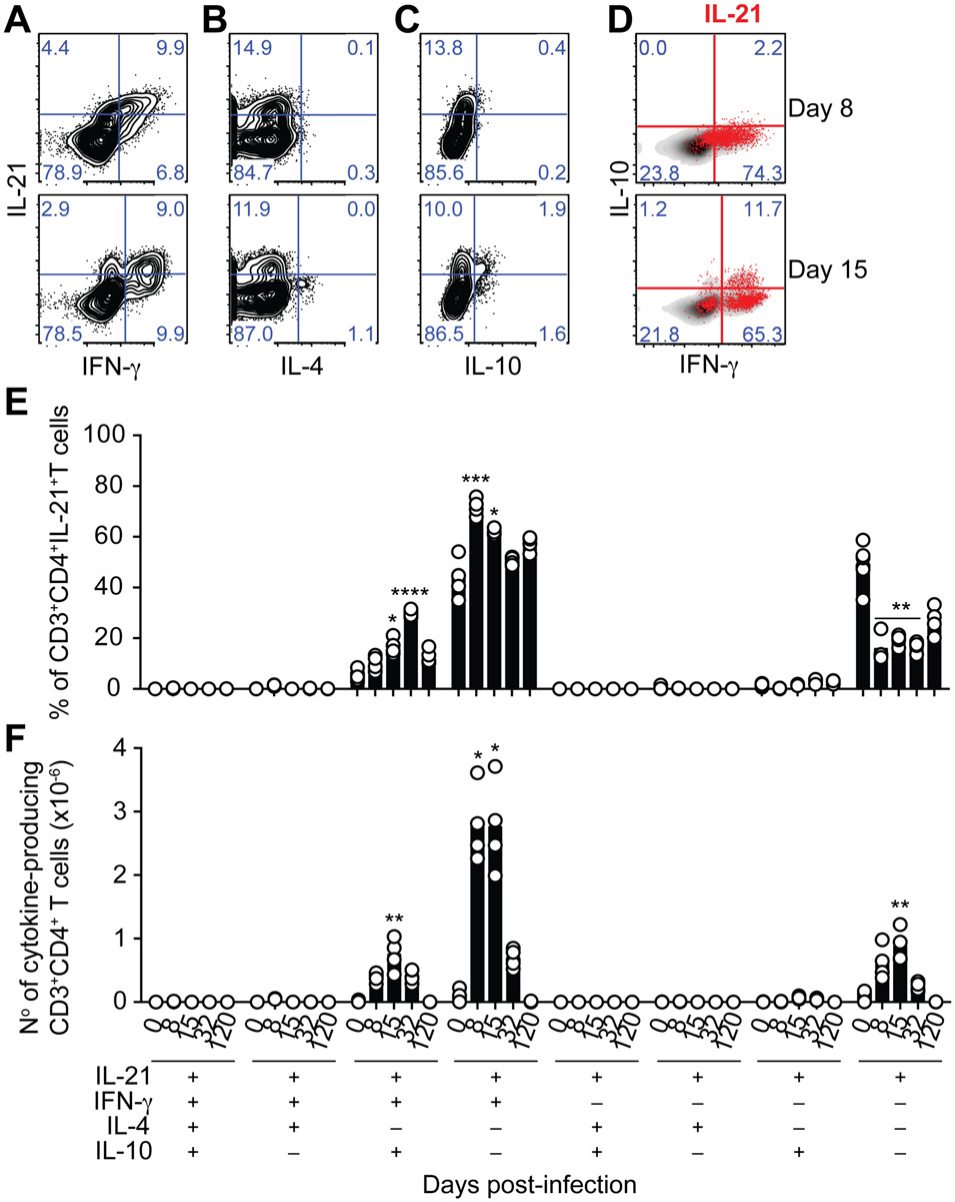
IL-21 is co-expressed with IFN-γ and IL-10 during *P*. *chabaudi* infection. (A-C) Flow cytometry plots showing individual examples for days 8 and 15 post-infection of different cytokine combinations studied in CD3^+^CD4^+^ T cells from the spleen of WT C57BL/6 mice. (D) IL-21-producing CD4^+^ T cells (red) overlaid on the plots corresponding to IFN-γ vs IL-10 on gated CD3^+^CD4^+^ T cells. Cumulative data showing the percentage (E) and total numbers (F) of IL-21-producing CD4^+^ T cells co-expressing IFN-γ, IL-4 and IL-10 in the spleen of WT C57BL/6 mice. The differential combination of expression (+) or absence of expression (–) of each cytokine (indicated in the bottom left) is shown for each subset at different days post-infection. Data are representative of at least two independent experiments and were obtained in groups of 4–6 mice per time point. Statistical significance was obtained using the Kruskal-Wallis test comparing each time point, corresponding to each cytokine combination with its respective basal level (day 0 post-infection). *, P<0.05; **, P<0.01; ***, P<0.001; ****, P<0.0001. Bars represent median values.

The majority of IL-21-producing CD4^+^ T cells in the spleens of WT C57BL/6 mice co-expressed IFN-γ; in particular at the peak of infection, when over 70% of the IL-21-producing CD4^+^ T cells in the spleen also expressed IFN-γ (Fig 3, A, E and F). The total number of CD4^+^ T cells co-expressing IL-21 and IFN-γ showed a dramatic increase by day 8 post-infection, remained high at day 15 post-infection, and decreased thereafter (Fig 3 F). Interestingly, some of the IL-21-producing CD4^+^ T cells expressing IFN-γ also expressed IL-10 (Fig 3, C-F). The frequency of these triple producers increased with the progression of the infection until day 32 post-infection, when a maximum of approximately 30% of IL-21-producing CD4^+^ T cells co-expressed IFN-γ and IL-10 (Fig 3 D and E). The highest number of IL-21-producing CD4^+^ T cells co-expressing IFN-γ and IL-10 was detected at day 15 post-infection (Fig 3 F). By day 120–140 post-infection, the pattern of cytokines co-expressed with IL-21 resembled that of naïve WT C57BL/6 mice (Fig 3, E and F). Altogether, these data show that CD4^+^ T cells are the main source of IL-21 in the spleens of WT C57BL/6 mice during *P*. *chabaudi* infection, and demonstrate the occurrence of multifunctional CD4^+^ T cells co-expressing IFN-γ and IL-10 together with IL-21.

**Fig 4:**
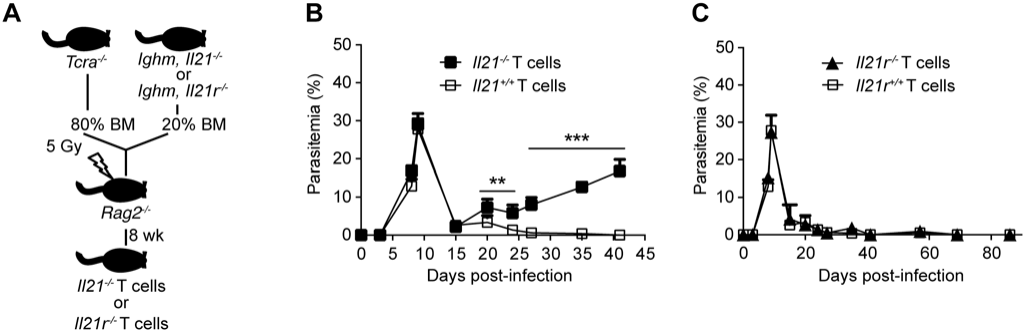
Mice bearing T cells deficient in IL-21 fail to control chronic *P*. *chabaudi* infection. Course of a *P*. *chabaudi* infection in mixed BM chimeric mice generated as described with the scheme in (A) and detailed in Materials and Methods and S1 Table, (B) with fully functional B cells and T cells deficient in the *Il21* gene (*Il21* ^*-/-*^ T cells, closed squares), and (C) with fully functional B cells and T cells deficient in the *Il21r* gene (*Il21r* ^*-/-*^ T cells, closed triangles) infected with *P*. *chabaudi*. As controls, mixed BM chimeric mice with BM from *Tcra* ^*-/-*^ and *Ighm* mice were generated (*Il21* ^*+/+*^ and *Il21r* ^*+/+*^ T cells, open squares, details in S1 Table). Statistical significance was obtained using Mann Whitney U test. **, P<0.01; ***, P<0.001. The graphs show the mean ± SEM of the parasitemia at different time points in 7–10 mice per group. Data are representative of two independent experiments.

Having identified CD4^+^ T cells as the producers of IL-21 during *P*. *chabaudi* infection, we wanted to confirm that IL-21 production by T cells was necessary to control *P*. *chabaudi* infection. To this end, we generated mixed bone marrow (BM) chimeras in which the deletion of the *Il21* gene, or its receptor, was restricted to T cells (Fig 4 A). Control groups consisted of mixed BM chimeric mice reconstituted with BM obtained from WT C57BL/6 mice, and from *Tcra* ^*-/-*^ [28] and *Ighm* [29] mice mixed in an 80:20 ratio. Similar to *Il21* ^*-/-*^ and *Il21r* ^*-/-*^ mice, mixed BM chimeric mice bearing *Il21* ^*-/-*^ T cells failed to control the chronic phase of infection and showed increasingly and sustained high parasitemias during this phase of infection (Fig 4 B). IL-21R signaling on T cells was not required to control *P*. *chabaudi* infection, as mixed BM chimeric mice bearing *Il21r* ^*-/-*^ T cells showed a normal course of *P*. *chabaudi* infection when compared with WT C57BL/6 mice or mixed BM chimeric control groups (Fig 4 C). Together, these data demonstrate the requirement for IL-21 production by CD4^+^ T cells to control chronic *P*. *chabaudi* infection.

### *P*. *chabaudi* infection promotes a robust Tfh cell response, even in the absence of IL-21 signaling

Tfh cells collaborate with B cells and are considered to be critical for the development of antigen-specific B cell responses during GC reactions [9, 10]. Tfh cells are a major source of IL-21, and this cytokine has been shown to be important for Tfh cell functionality. Therefore, we assessed the numbers of Tfh cells generated during a *P*. *chabaudi* infection, whether these cells were generated, whether they were the source of IL-21, and whether IL-21 was required for their generation and maintenance.

**Fig 5:**
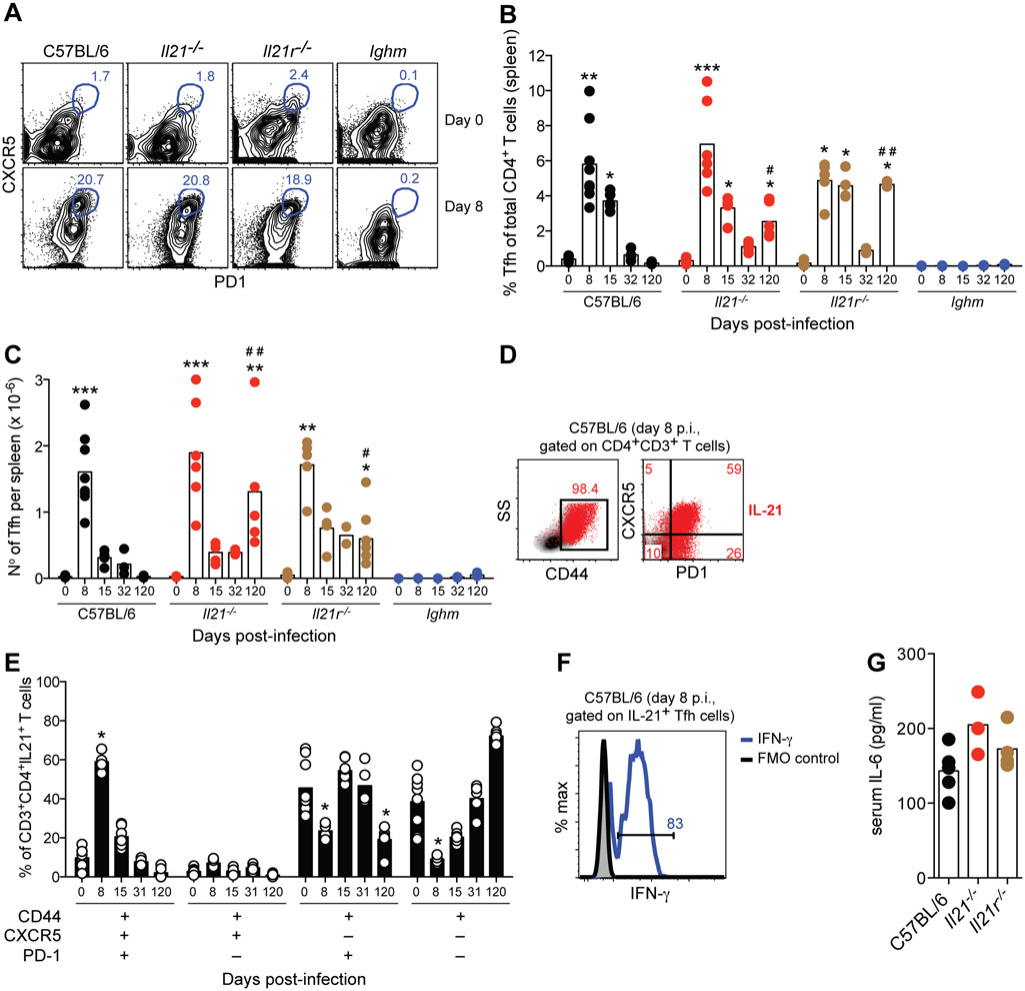
IL-21-producing Tfh cells are activated during acute *P*. *chabaudi* infection. (A) Flow cytometric analysis of representative naïve (top row) and infected mice (8 days post-infection, bottom row). Gates show frequency of CD3^+^CD4^+^CD44^high^ cells expressing CXCR5 and PD-1. (B) Frequency and (C) total numbers of Tfh cells, defined as CD3^+^CD4^+^CD44^high^CXCR5^+^PD-1^+^, in WT C57BL/6, *Il21* ^*-/-*^, *Il21r* ^*-/-*^ and *Ighm* mice. (D) Flow cytometric analysis representative of infected WT C57BL/6 mice (8 days post-infection) corresponding to IL-21 intracellular staining on CD4^+^ T cells (red), overlaid on side scatter light vs CD44 (left) and CXCR5 vs PD-1 (right) from CD3^+^CD4^+^ T cells. Numbers show frequency of IL-21-producing CD4^+^ T cells with high expression of CD44 (left), and their differential expression of CXCR5 and PD-1 (right). (E) Differential combination of expression (+) or absence of expression (–) of CD44, CXCR5 and PD-1 (bottom left) on IL-21-producing CD3^+^CD4^+^ T cells at different days post-infection in the spleen of WT C57BL/6 mice. (F) Flow cytometric analysis of IFN-γ (green line) on CD3^+^CD4^+^CD44^high^CXCR5^+^PD-1^+^IL-21^+^ T cells from the spleen of WT C57BL/6 mice, 8 days post-*P*. *chabaudi* infection (representative of 4 mice). (G) Serum IL-6 at day 6 post-*P*. *chabaudi* infection. Statistical significance was obtained using the Kruskal-Wallis test comparing each time point with its respective basal level (day 0 post-infection) (*, P<0.05; **, P<0.01; ***, P<0.001), or comparing with the data obtained from the WT C57BL/6 group (#, P<0.05; # #, P<0.01). Bars represent median values. Data are representative of at least two independent experiments and were obtained in groups of 4–7 mice per time point.

For multiparameter flow cytometry analysis, we defined Tfh cells as CD3^+^CD4^+^CD44^high^CXCR5^+^PD-1^+^ (S3A Fig), and confirmed the identity of Tfh cells by intranuclear staining of the master regulator of Tfh cell differentiation, the transcription factor Bcl-6 (S3B Fig). Infection of WT C57BL/6 mice with *P*. *chabaudi* led to an increase in Tfh cells, as evinced by a greater than 14-fold and 60-fold increase in their frequency and total numbers, respectively, in the spleen, 8 days post-infection, when compared to basal levels (Fig 5, A-C). At the peak of *P*. *chabaudi* infection, approximately 60% of the IL-21-producing CD4^+^ T cells in the spleen of WT C57BL/6 mice showed a Tfh cell phenotype (Fig 5, D and E). The majority of the IL-21-producing Tfh cells at the peak of *P*. *chabaudi* infection co-expressed IFN-γ (74.3±1.3%, Fig 5 F). Both the frequency and total numbers of Tfh cells in the spleen decreased at day 15 post-infection, reached basal levels by day 32 post-infection, and remained low at later time points of the study (Fig 5, A-C).

Neither IL-21, nor IL-21R, was required to generate a Tfh cell response during *P*. *chabaudi* infection, as the kinetics and magnitude of Tfh cell responses in *Il21* ^*-/-*^ and *Il21r* ^*-/-*^ was essentially similar to those observed in WT C57BL/6 mice (Fig 5, A-C). As IL-6 has also been implicated in Tfh differentiation [9], we determined whether infected *Il21* ^*-/-*^ and *Il21r* ^*-/-*^ mice could produce IL-6, which might explain their ability to generate Tfh cells. In both knockout strains, IL-6 was detected in the plasma at day 6 of infection at levels not significantly different from those of infected WT C57BL/6 controls (Fig 5 G). The slightly higher numbers of Tfh cells in *Il21* ^*-/-*^ and *Il21r* ^*-/-*^ at day 120 post-infection compared with those in WT C57BL/6 mice might have been a consequence of the on-going infection promoting continued T cell activation. In accordance with this idea, both *Il21* ^*-/-*^ and *Il21r* ^*-/-*^ mice showed higher frequencies of CD44^high^ and PD-1^+^ CD4^+^ T cells in the spleen at day 120 post-infection when compared to WT C57BL/6 mice (frequencies CD4^+^CD44^high^ of 82±5% and 62±14% for *Il21* ^*-/-*^ and *Il21r* ^*-/-*^ vs 14±2% for WT C57BL/6; frequencies CD4^+^PD-1^+^ of 74±5 and 61±7 for *Il21* ^*-/-*^ and *Il21r* ^*-/-*^ vs 8±2 for WT C57BL/6; P<0.01, Kruskal-Wallis test). *P*. *chabaudi* infection failed to activate Tfh cells in B-cell-deficient *Ighm* mice (Fig 5, A-C), which is in agreement with previous findings showing a requirement for the presence of B cells [30] and direct interactions with B cells [31] for the activation of Tfh cells.

These data show a strong activation of Tfh cell responses during acute *P*. *chabaudi* infection, which is not affected by the lack of signaling through the IL-21R. This T cell subset represents an important source of IL-21 at the peak of *P*. *chabaudi* infection, and co-expresses IFN-γ.

### IL-21 is required to generate *P*. *chabaudi*-specific B cell responses, and is necessary for protective immunity against a secondary challenge infection

As IL-21 signaling is required to control the chronic phase of *P*. *chabaudi* infection and Tfh cells are an important source of IL-21, we reasoned that the lack of IL-21 signaling would impair the development of protective B cell responses and consequently prevent the resolution of the infection.

**Fig 6:**
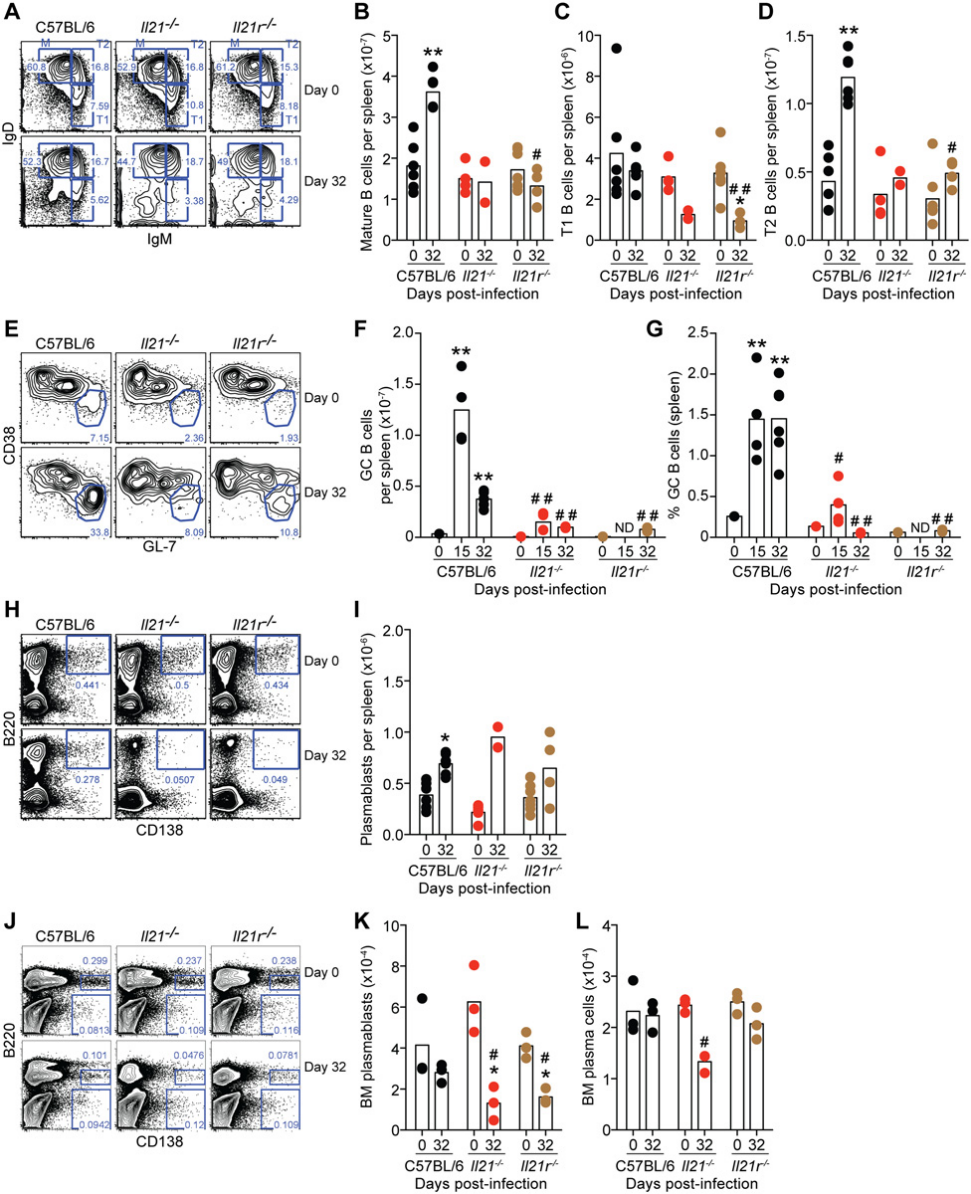
Flow cytometry analysis of B cell responses during *P*. *chabaudi* infection in WT C57BL/6, *Il21* ^*-/-*^ and *Il21r* ^*-/-*^ mice. (A-D) Analysis of Mature (M) Transitional 1 (T1) and Transitional 2 (T2) on CD19^+^B220^+^ gated B cells in the spleen based on the pattern of IgD and IgM expression. (E-G) Analysis of GL-7^high^CD38^low^ GC cells on CD19^+^IgD^–^ gated B cells in the spleen. (H-I) Analysis of B220^+^CD138^+^ plasmablasts in the spleen. (J-L) Analysis of B220^+^CD138^high^ plasmablasts and B220^–^CD138^high^ plasma cells in the BM (1 femur plus 1 tibia per mouse). Statistical significance was obtained using the Kruskal-Wallis test comparing each time point with its respective basal level (day 0 post-infection) (*, P<0.05; **, P<0.01), or comparing with the data obtained from the WT C57BL/6 group (#, P<0.05; # #, P<0.01). The Mann Whitney U test was used to compare with its respective basal level when sets of data of only 2 time points were available (*, P<0.05). Bars represent median values. Data are representative of at least two independent experiments and were pooled from groups of 3–4 mice per time point.

Flow cytometric analysis of the different B cell compartments in spleen and BM showed no significant differences in naïve *Il21* ^*-/-*^ and *Il21r* ^*-/-*^ mice compared with those of naïve WT C57BL/6 controls (Fig 6). At day 32 post-infection, the numbers of B cells in the spleen of *Il21* ^*-/-*^ and *Il21r* ^*-/-*^ mice were reduced compared to WT C57BL/6 controls (S2 Fig), and this was reflected in the numbers of mature (M), transitional 1 (T1) and transitional 2 (T2) B cells (Fig 6, A-D). A robust CD19^+^IgD^–^GL-7^high^CD38^low^ GC B cell response in the spleen of WT C57BL/6 controls was observed at day 15 post-infection and sustained at day 32 post-infection (Fig 6, E-G). In stark contrast, both *Il21* ^*-/-*^ and *Il21r* ^*-/-*^ mice failed to generate this GC response during *P*. *chabaudi* infection (Fig 6, E-G). The numbers of B220^+^CD138^+^ plasmablasts in the spleen of WT C57BL/6 controls were increased at day 32 post-infection (Fig 6, H and I). Interestingly, the numbers of B220^+^CD138^+^ plasmablasts in the spleen of *Il21* ^*-/-*^ and *Il21r* ^*-/-*^ mice were similar to those of WT C57BL/6 mice (Fig 6, H and I). However, the numbers of B220^+^CD138^high^ plasmablasts in BM from *Il21* ^*-/-*^ and *Il21r* ^*-/-*^ mice, and the numbers of B220^–^CD138^high^ plasma cells in BM from *Il21* ^*-/-*^ mice were reduced at day 32 post-infection compared to those of WT C57BL/6 controls (Fig 6, J-L).

**Fig 7:**
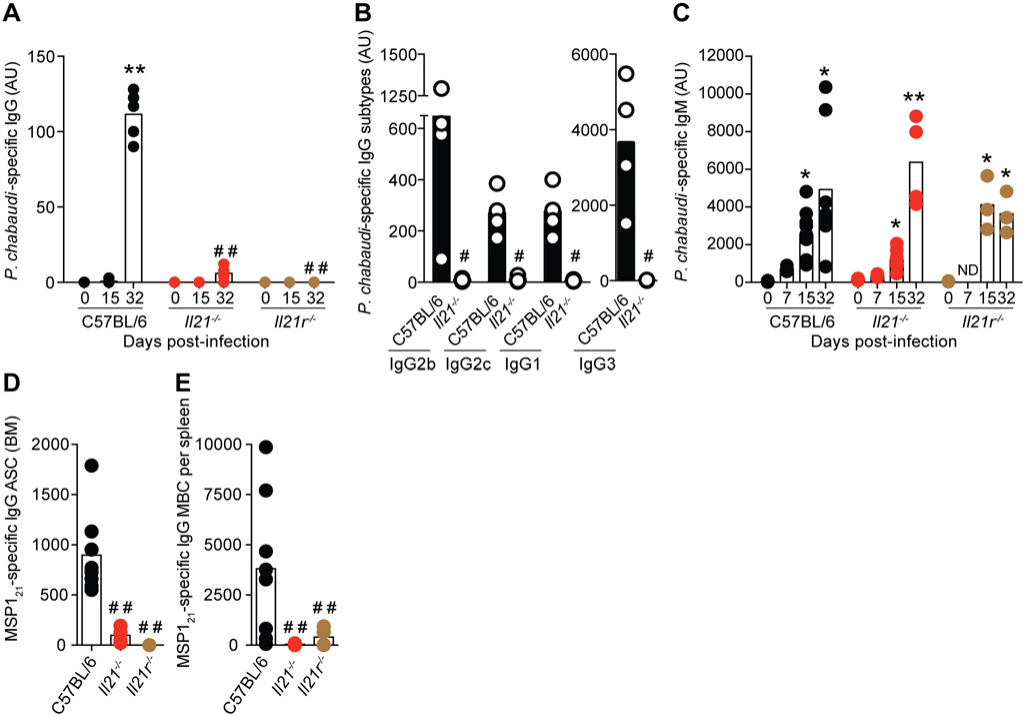
*P*. *chabaudi*-specific IgG B-cell responses are abrogated in the absence of IL-21 signaling. (A) IgG, (B) IgG subtypes (day 32 post-infection) and (C) IgM antibodies specific for a lysate of *P*. *chabaudi*-infected rbc determined by ELISA. Antibody units (AU) were calculated based on the *P*. *chabaudi*-specific antibody levels of a hyper-immune standard plasma defined as 1000 U. In the cases where levels of antibodies were below background, arbitrary values of 2 log lower than the mean value observed in WT C57BL/6 mice were set to be able to perform the statistical test. (D) MSP1_21_-specific IgG-producing ASC in BM obtained from one femur and one tibia, and (E) MBC per spleen, determined by ELISPOT 32 days post-infection. Statistical significance was obtained using the Kruskal-Wallis test comparing each time point with its respective basal level (day 0 post-infection) (*, P<0.05; **, P<0.01), or comparing with the data obtained from the WT C57BL/6 group (# #, P<0.01). The Mann Whitney U test was used in the case of IgG subtypes, comparing *Il21* ^*-/-*^ vs WT C57BL/6 mice (#, P<0.05). Bars represent median values. Data are representative of at least two independent experiments and were obtained in groups of 3–8 mice per time point.

Although there were no differences in the initial numbers of B cells, *P*. *chabaudi*-specific IgG antibodies of all isotypes were absent in both *Il21* ^*-/-*^ and *Il21r* ^*-/-*^ mice at all evaluated time points (Fig 7, A and B). IgM antibodies, on the other hand, were not significantly affected by the lack of IL-21 signaling (Fig 7 C). In line with the absence of IgG antibodies, there was also a loss of both *P*. *chabaudi*-specific IgG antibody-secreting-cells (ASC) in the BM (Fig 7 D), and of *P*. *chabaudi*-specific IgG MBC in the spleen (Fig 7 E) at day 32 post-infection in both *Il21* ^*-/-*^ and *Il21r* ^*-/-*^ mice when compared with WT C57BL/6 controls.

**Fig 8:**
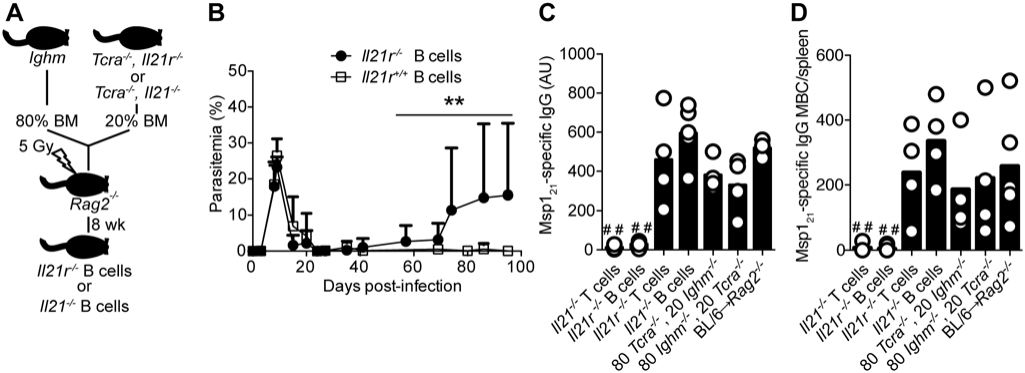
Mice bearing IL-21R-deficient B cells fail controlling chronic *P*. *chabaudi* infection and activating *P*. *chabaudi*-specific-IgG-responses. (A) Scheme describing the approach applied to generate mice with fully functional T cells and B cells deficient in the *Il21* or the *Il21r* gene (details in Materials and Methods and S1 Table). (B) Course of a *P*. *chabaudi* infection in mixed BM chimeric mice with B cells deficient in the *Il21r* gene (*Il21r* ^*-/-*^ B cells, closed circles); as controls, mixed BM chimeric mice with BM from *Tcra* ^*-/-*^ and *Ighm* mice were generated (*Il21r* ^*+/+*^ B cells, open squares, details in S1 Table). Statistical significance was obtained using the Mann Whitney U test (**, P<0.01). The graph shows the mean ± SEM of the parasitemia at different time points in 7–10 mice per group. (C) MSP1_21_-specific IgG antibodies determined by ELISA 32 days post-infection in different mixed BM chimeric groups (4–5 mice per group, details in S1 Table). (D) Total number of MSP1_21_-specific IgG MBC in spleens from different mixed BM chimeric groups, determined by ELISPOT on days 120–150 post-infection (4–5 mice per group, details in S1 Table). Statistical significance was obtained using the Kruskal-Wallis test comparing the data obtained from the group of *Rag2* ^*-/-*^ mice reconstituted with BM from WT C57BL/6 mice (BL/6→ *Rag2* ^*-/-*^. # #, P<0.01). Bars represent median values. Data are representative of two independent experiments.

To evaluate whether IL-21 directly signaled B cells to mount an antibody response, and thus to resolve the chronic phase of *P*. *chabaudi* infection, we generated mixed BM chimeric mice in which a targeted deletion of the *Il21r* gene was restricted to B cells (Fig 8 A). Control groups consisted of mixed BM chimeric mice reconstituted with BM obtained from WT C57BL/6 mice, and from *Tcra* ^*-/-*^ [28] and *Ighm* [29] mice mixed in a 20:80 ratio. Similar to the *Il21r* ^*-/-*^ mice, the mixed BM chimeric mice bearing *Il21r* ^*-/-*^ B cells eventually developed a non-resolving chronic-stage parasitemia (Fig 8 B). Furthermore, the mixed BM chimeric mice bearing *Il21r* ^*-/-*^ B cells, as well as the mixed BM chimeric mice bearing *Il21* ^*-/-*^ T cells, but not those bearing *Il21r* ^*-/-*^ T cells or *Il21* ^*-/-*^ B cell, showed a dramatic decrease in the levels of *P*. *chabaudi*-specific IgG antibodies and in the numbers of *P*. *chabaudi*-specific IgG MBC in the spleen (Fig 8, C and D).

**Fig 9:**
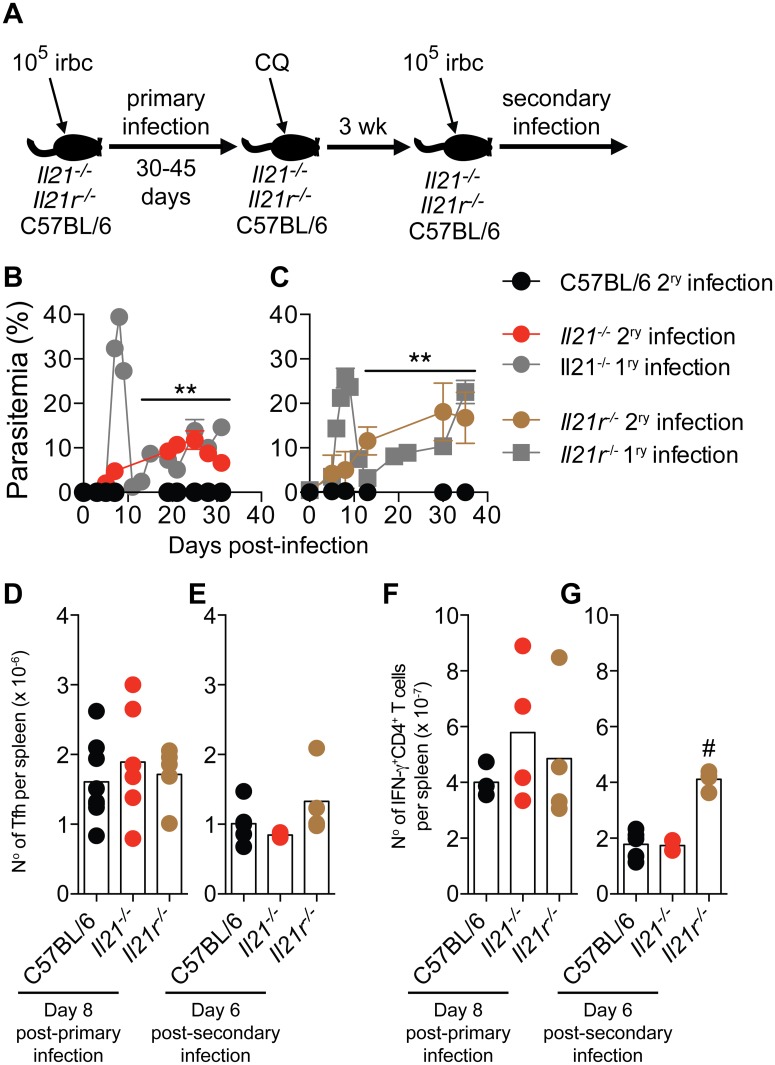
Mice deficient in IL-21 signaling fail to develop immunity to a secondary *P*. *chabaudi* infection. (A) Scheme describing the experimental approach. CQ = chloroquine. (B and C) *P*. *chabaudi*-infected mice were treated with chloroquine to eliminate parasitemia as described in the materials and methods, and re-infected with 10^5^ *P*. *chabaudi-*infected rbc (day 0 post-secondary infection). The graphs show the course of secondary *P*. *chabaudi* infection in WT C57BL/6 (black circles), *Il21* ^*-/-*^ (red circles) and *Il21r* ^*-/-*^ (brown circles) mice; course of primary infection in *Il21* ^*-/-*^ (gray circles) and *Il21r* ^*-/-*^ (gray squares) are overlaid. (D and E) Number of Tfh cells per spleen post-primary and secondary infection, respectively. (F and G) Number of IFN-γ^+^CD4^+^ T cells per spleen post-primary and secondary infection, respectively. Data are representative of two independent experiments and are obtained in groups of 3–10 mice per time point. Statistical significance was obtained using Mann Whitney U test (**, P<0.01) or Kruskal-Wallis test (#, P<0.05). Error bars correspond to mean ± SEM.

C57BL/6 mice develop significant immunity to a second infection with the same strain of *P*. *chabaudi* and are able control the challenge infection at very low parasitemias [27]. Because infected *Il21* ^*-/-*^ and *Il21r* ^*-/-*^ mice cannot make *P*. *chabaudi*-specific IgG responses, and in particular MBC responses, we reasoned that lack of IL-21 signaling would render these mice unable to control a re-infection. We therefore infected *Il21* ^*-/-*^, *Il21r* ^*-/-*^ and WT C57BL/6 mice with 10^5^ *P*. *chabaudi*-irbc, treated them with chloroquine 30–45 days post-infection to eliminate the chronic primary infection, and re-challenged them with 10^5^ *P*. *chabaudi*-irbc of the same strain 3 weeks after drug treatment (Fig 9 A). In accordance with our previous data, WT C57BL/6 similarly treated with chloroquine during the primary chronic infection were immune to a second challenge with very low parasitemias. In stark contrast, both *Il21* ^*-/-*^ and *Il21r* ^*-/-*^ mice failed to resolve the second infection and showed sustained high parasitemias (Fig 9, B and C). Interestingly, neither *Il21* ^*-/-*^ nor *Il21r* ^*-/-*^ mice showed the peak of parasitemia characteristic of an acute primary infection after receiving the second *P*. *chabaudi* challenge, suggesting that some initial immune control could take place in the absence of an IgG B-cell response (Fig 9, B and C). Similarly, *Il21r* ^*-/-*^ mice also failed to resolve a second infection with *P*. *yoelii* 17X(NL), in contrast to WT C57BL/6 controls, which controlled parasitemias at very low levels (S1B Fig).

Similar to Tfh responses during primary infection (Fig 5 A-C and Fig 9 D), Tfh responses during second *P*. *chabaudi* infection (Fig 9 E) and IFN-γ responses of CD4^+^ T cells during primary and second *P*. *chabaudi* infection (Fig 9 F and G) were not altered by the absence of IL-21 signaling.

These data show that IL-21 is necessary to activate *P*. *chabaudi*-specific IgG B-cell responses, by direct signaling through the IL-21R on B cells, thus resolving the chronic infection. Moreover, IL-21 signaling is required to activate *P*. *chabaudi*-specific IgG MBC responses, and to develop immunity to homologous secondary *P*. *chabaudi* and *P*. *yoelii* 17X(NL) infections.

## Discussion

Here we demonstrate, for the first time, a direct requirement of IL-21 signaling on B cells for the elimination of a systemic infection, and its importance in the control of chronic malaria. In the model of *P*. *chabaudi* infection in C57BL/6 mice, IL-21 produced by CD4^+^ T cells, predominantly Tfh cells, during the acute phase of an erythrocytic-stage *P*. *chabaudi* infection, is necessary for development of ASC, specific IgG antibodies and MBC, and to bring about the resolution of a chronic *P*. *chabaudi* infection. In a similar way, IL-21 signaling was required to control and eliminate a *P*. *yoelii* 17X(NL) infection. Our data highlight, for the first time, the importance of IL-21-dependent B-cell responses in the acquisition of immunity to re-infection and suggest that long-lived B-cell responses are required to achieve immunity to malaria re-infection.

Both *Il21* ^*-/-*^ and *Il21r* ^*-/-*^ mice failed to produce *P*. *chabaudi*-specific IgG antibodies, and did not generate *P*. *chabaudi*-specific IgG-producing MBC in the spleen or ASC in the bone marrow showing that IL-21 is required for class switching, and demonstrating that class switching to IgG responses is required for effective control of a *P*. *chabaudi* infection. *P*. *chabaudi*-specific IgM responses were not altered in the absence of IL-21 signaling. IgM can be produced by short-lived plasmablasts in the spleen, which have not undergone development in GC [32], perhaps explaining why total plasmablast numbers in the spleen were unaffected by the lack of IL-21 signaling. As we show that IL-21 signaling is required to activate GC B-cell responses upon *P*. *chabaudi* infection, we believe that IL-21 signaling is required to activate early stages of T-dependent B-cell responses to the parasites. However, we cannot rule out a direct requirement of IL-21 in the signal pathways leading to class switch recombination and MBC generation. As mixed BM chimeric mice with B cells deficient in IL-21R showed impairment of the humoral response, and did not resolve the chronic phase of *P*. *chabaudi* infection, it appears that there is a direct requirement for IL-21 from T cells to signal through the IL-21R on B cells to induce the differentiation of B cells into plasma cells, and thereby for antibody production. Our data is in accordance with those studies showing the critical importance of IL-21 signaling in IgG responses following immunization [11, 15, 23], but contrasts with studies of other pathogens, where there are variable requirements for IL-21 signaling for B-cell responses. In LCMV- and Influenza-infected mice, the lack of IL-21 signaling results only in slight, or no alterations in induction of B-cell responses, but the IgG antibody response is poorly sustained [19, 22, 25, 26]. Despite decreased levels of IgG1, isotype-switching and GC formation are not altered in *Il21r* ^*-/-*^ mice infected with *H*. *polygyrus* [14]. Primary B-cell responses, but not MBC responses, against live rabies virus-based vaccines, require IL-21 signaling [12]. The reasons for these differential requirements for IL-21 in the different infections/immunizations are not known. It is possible that other signals such as co-stimulation via TLR ligation, cytokines like IL-4, or strength of the BCR signal through extensive BCR cross-linking, could partially compensate for the lack of IL-21 signaling. Intrinsic differences in the antigens and co-stimulations delivered by different infections or immunizations could then differentially engage signals able to compensate different aspects of the B-cell response that would otherwise require signaling through IL-21. In this regard, signaling through TLR7 is known to be able to restore defective B-cell activation in *Il21r* ^*-/-*^ mice [11].

The impaired control of parasitemia and loss of the humoral response to *P*. *chabaudi* in *Il21* ^*-/-*^ and *Il21r* ^*-/-*^ mice were not due to lack of development of phenotypically-defined Tfh cells. Mice lacking IL-21 or the IL-21R in all cells were able to generate WT levels of Tfh cells; and mice lacking IL-21R only in T cells also had specific B-cell responses similar to those of WT mice. The requirement of IL-21 for the generation or maintenance of Tfh cell responses is controversial. Some studies have shown that IL-21 signaling is required for development, maintenance, or functional competence [15, 16, 19–21]. However, similar to our studies, others have shown that Tfh cell responses are normal or even elevated in the complete absence of IL-21 signaling [11–14, 17, 18, 22, 23]. Thus, the requirement for IL-21 to activate Tfh responses seems to be highly dependent on the model of immunization or infection studied. In this *P*. *chabaudi* infection, direct IL-21 signaling on T cells is not required to activate a functional Tfh program. It is possible that IL-6, which we show here is produced at levels similar to those of infected WT mice, compensates for the lack of IL-21 in the generation of Tfh cells, as has been described [13, 16, 33, 34].

CD8^+^ T cells have been shown to require IL-21 for protective responses in chronic viral infections in humans and mice [22, 25, 26, 35–37], and are known to play some role in controlling erythrocytic-stage *Plasmodium* infections in mice [38–42]. Our data rule out a mechanism by which IL-21 acts on or via CD8^+^ T cells to resolve the chronic *P*. *chabaudi* infection, since, in mixed BM chimeric mice in which there is a deficiency of IL-21R only in T cells, there was no exacerbation of the chronic infection.

Despite the failure of *Il21* ^*-/-*^ and *Il21r* ^*-/-*^ mice to resolve their chronic infection, they did not succumb to a fulminating parasitemia within the 100–150 days of the study. One possible factor that may limit the chronic parasitemia is the preference of *P*. *chabaudi* for mature rbc [43]. The presence of large numbers of Ter119^+^ rbc in the spleens of *Il21* ^*-/-*^ and *Il21r* ^*-/-*^ mice indicates increased hematopoiesis in response to anemia, presumably leading to production of many new rbc, which then controls the level of parasitemia. In addition, there are likely to be numerous B-cell-independent innate effector mechanisms activated, which could partially control parasitemia, but unable to completely clear the infection by themselves. Different from infections in *Il21* ^*-/-*^ and *Il21r* ^*-/-*^ mice, mixed BM chimeric mice with B cells deficient in IL-21R did not show a significant difference in relapsing parasitemia compared to WT C57BL/6 mice until days 70–80 post-infection. The higher variability in the parasitemias in these mixed BM chimeric mice compared to *Il21r* ^*-/-*^ late in infection may reflect B-cell-independent alternate mechanisms, which require IL-21. In general agreement with this idea, IL-21 has been shown to be involved in activation of macrophages and NK cells [44], both cell types implicated in the control of *Plasmodium* infection [45].

Previous studies have shown that Th1, Th2 and Th17 CD4^+^ T cell subsets can also produce IL-21 [9], and that Tfh cells can express cytokines characteristic of Th1/Th2/Th17 subsets [46]. This suggests either that other subsets of CD4^+^ T cells can produce IL-21, or that Th1/Th2/Th17 subsets activated in infections can acquire an additional Tfh phenotype. The generation of different CD4^+^ T cell subsets during infections is a very dynamic process, and activated CD4^+^ T cells, including Tfh cells, show substantial plasticity with overlapping phenotypes [47]. In the case of *P*. *chabaudi* infection, IL-21 production by CD4^+^ T cells was strongly linked to that of IFN-γ, the hallmark cytokine of Th1 CD4^+^ T cells, reflecting the strong bias to a Th1 response during acute *P*. *chabaudi* infection. We found no IL-4/IL-21 double-producing cells, and there is little induction of IL-17 in the spleens of *P*. *chabaudi*-infected mice [48]. Later in the course of *P*. *chabaudi* infection (13–15 days), we observed the appearance of splenic CD4^+^ T cells co-expressing IL-21, IFN-γ and IL-10. IL-10 may have been induced in these cells in order to regulate the inflammatory response and thus immune-mediated pathology, as we have shown previously [49, 50].

IL-21-producing CD4^+^ T cells are present in peripheral blood mononuclear cells from malaria-exposed immune adults [51, 52] and correlate with *P*. *falciparum*-specific IgG antibodies in children with acute *falciparum* malaria [53]. These observations encourage us to believe that use of the mouse model of *P*. *chabaudi* is a valid approach to dissect the regulation and role of IL-21, Tfh and B-cell responses, which is also relevant to other infections dependent on humoral immunity for their elimination.

In summary, we show that the absence of IL-21 signaling on B cells results in a loss of capacity to activate *Plasmodium*-specific IgG antibodies and memory B cells, resulting in a failure to resolve a chronic erythrocytic-stage infection with *P*. *chabaudi* and an inability to control a secondary challenge infection. This important immune mechanism should receive particular attention when exploring novel vaccine strategies.

## Materials and Methods

### Ethics statement

All animal experiments were approved by the MRC NIMR institutional Ethical Review Panel and carried out according to UK National guidelines (Scientific Procedures) Act 1986 under license PPL80/2385 approved by the British Home Office.

### Mice

C57BL/6, *Ighm* [29], *Tcra* ^*-/-*^ [28] and *Rag2* ^*-/-*^ [54] mouse strains were bred in the specific pathogen-free facilities of the MRC NIMR and were backcrossed for at least 10 generations onto NIMR C57BL/6 mice. Mice with a targeted deletion of the *Il21* gene, originally from NIH MMRRC (F2 129/ SvEvBrd x C57BL6/J), were obtained from Manfred Kopf, Institute for Molecular Health Sciences, Zürich, Switzerland. They were backcrossed 3 generations to C57BL/6J at the Garvan Institute, Sydney, and then backcrossed for 5 generations by Manfred Kopf and a further 3 generations onto NIMR C57BL/6 mice. *Il21r* ^*-/-*^ mice backcrossed to C57BL/6 (N7) were a kind gift from Manfred Kopf [55], and were further backcrossed 3 times onto NIMR C57BL/6. In all experiments, NIMR C57BL/6 bred in the same animal house were used as controls. From a panel of 768 SNPs, NIMR C57BL/6 and C57BL/6/J mice differ only by 2 SNPs; one each on chromosomes 2 and 12.

### Mixed BM chimeras

The *Il21* ^*-/-*^ and *Il21r* ^*-/-*^ mice were crossed with *Ighm* and *Tcra* ^*-/-*^ to obtain double knockout strains *Ighm Il21* ^*-/-*^, *Ighm Il21r* ^*-/-*^, *Tcra* ^*-/-*^ *Il21* ^*-/-*^, and *Tcra* ^*-/-*^ *Il21r* ^*-/-*^. To generate mixed BM chimeric mice in which either B or T cells were deficient in the expression of IL-21 or IL-21R, femurs and tibias from donor mice were excised and cleaned of flesh using forceps and scalpel, and BM was obtained by flushing out with IMDM supplemented with 2 mM L-glutamine, 0.5 mM sodium pyruvate, 100 U penicillin, 100 mg streptomycin, 6 mM Hepes buffer, and 50 mM 2-ME (all from Gibco, Invitrogen), using a syringe with a needle. Thereafter, single BM cell suspensions were obtained by mashing through a 70 μm filter mesh, further sieved through 40 μm filter mesh and washed once. Live cells were resuspended in sodium chloride solution 0.9% (Sigma) at 4x10^6^cells/200 μl. *Rag2* ^*-/-*^ mice were sub-lethally irradiated (5 Gy) using a [^137^Cs] source and reconstituted less than 24hr after irradiation by i.v. injections of different combinations of donor BM cells (S1 Table and S4 Fig). Recipient mice were maintained on acidified drinking water and analyzed for reconstitution after 8 weeks. Only mixed BM chimeric mice showing frequencies of T and B cells similar to those of WT C57BL/6 control mice were included in experiments.

### Infection of mice with *P*. *chabaudi* and *P*. *yoelii*

Mice were infected with 10^5^ *P*. *chabaudi chabaudi AS*–infected or 10^3^ *P*. *yoelii* 17X(NL)-infected rbc by i.p. injections, and parasitemias monitored by Giemsa-stained blood films, as previously described [56]. The total number of rbc and hemoglobin concentrations were determined using a Vetscan (Abaxis), and body ventral temperature was measured using a MiniTemp MT6 infrared thermometer (Raytek).

### Drug treatment

Drug-mediated elimination of *P*. *chabaudi* infection in *Il21* ^*-/-*^, *Il21r* ^*-/-*^ and WT C57BL/6 mice was accomplished with 10 i.p. injections of 40 mg chloroquine (Sigma)/kg body weight in 0.9% sodium chloride solution given in 10 consecutive days, starting on day 30–45 post-primary infection. Drug-mediated elimination of *P*. *yoelii* in *Il21r* ^*-/-*^ and WT C57BL/6 was accomplished with chloroquine given in drinking water (600mg/L) during 6 consecutive days, followed by 3 i.p. injections of 0.25 mg pyrimethamine (Sigma) given in 3 consecutive days, followed by 3 i.p. injections of 1.25 mg of artesunat (Pharbaco) given in 3 consecutive days. Elimination of parasitemia was monitored by Giemsa-stained blood films and, in the case of *P*. *chabaudi* infection, to verify complete elimination of parasitemia after chloroquine treatment, 50 μl of blood were obtained from each mouse, diluted in 350 μl Kreb’s saline containing glucose (11 mM) and heparin (50 mU) and subinoculated into *Rag2* ^*-/-*^ mice. Giemsa-stained thin blood films from the recipient *Rag2* ^*-/-*^ mice were monitored for 3 weeks [27]. A second infection of 10^5^ *P*. *yoelii*-infected rbc was given to *Il21r* ^*-/-*^and WT C57BL/6 control mice 3 days after the last artesunat injection.

### Quantitative real-time PCR

Expression of IL-21 mRNA was measured as previously described [48]. Briefly, total RNA was extracted from splenocytes from uninfected and *P*. *chabaudi*-infected C57BL/6 mice with TRIzol reagent (Life Technologies), reverse transcribed, and IL-21 mRNA was measured using real-time quantitative PCR with the primers, forward TCATCATTGACCTCGTGGCCC and reverse ATCGTACTTCTCCACTTGCAATCCC.

### Flow cytometry

Spleens were dissected and single cell suspension was obtained by mashing the organs through a 70 μm filter mesh in HBSS (Gibco, Invitrogen). After removal of rbc by treatment with lysing buffer (Sigma), the remaining cells were resuspended in complete IMDM [IMDM supplemented with 10% FBS Serum Gold (PAA Laboratories, GE Healthcare), 2 mM L-glutamine, 0.5 mM sodium pyruvate, 100 U penicillin, 100 mg streptomycin, 6 mM Hepes buffer, and 50 mM 2-ME (all from Gibco, Invitrogen)] and viable cells were counted using trypan blue exclusion (Sigma) and a hemocytometer. Cells were then resuspended in PBS containing 2% FBS, 0.1% N_a_N_3_ (staining buffer), with the monoclonal anti-mouse CD16/32 [24G2, [57]] to block Fc-mediated binding of antibodies. To identify Tfh cells, 2x10^6^ cells were first incubated with biotin anti-CXCR5 in complete IMDM (BD Pharmingen), washed twice with staining buffer, resuspended in PBS and incubated with appropriate dilutions of PE or APC streptavidin, PE/Cy7 anti-PD-1, APC/Cy7 anti-CD4, FITC or PerCP/Cy5.5 anti-CD44 and APC or Pacific Blue anti-CD3e (Biolegend). Cells were fixed with 2% paraformaldehyde and stored in staining buffer at 4°C until acquisition. For B cell analysis, spleens and BM (1 femur plus 1 tibia) were prepared as described above, and 2x10^6^ cells were first incubated with anti-mouse CD16/32, followed by surface staining with different combinations of BV605 or BV785 anti-CD19, APC or PE/Cy7 anti-B220, BV421 anti-IgD, PerCP/Cy5.5 anti-IgM (all from Biolegend), FITC anti-GL-7, PE anti-CD138 (BD Pharmingen) and APC anti-CD38 (eBioscience), and acquired after two washes with PBS. To identify the different cell populations in the spleen, 2x10^6^ cells were first incubated with anti-mouse CD16/32, followed by surface staining with different combinations of BV785 anti-CD19, BV711 anti-NK1.1, BV650 anti-CD8, BV605 anti-CD4, BV421 anti-γδTCR, PerCP/Cy5.5 anti-Ly6G, FITC anti-MHCII, PE anti-Ly6C, APC/Cy7 anti-Ter119, alexa fluor 700 anti-CD3 and alexa fluor 647 anti-CD11c (all from Biolegend), and acquired after two washes with PBS.

For intracellular cytokine staining, cells were stimulated for 5h in complete IMDM with PMA (50 ng/mL; Sigma), Ionomycin (500 ng/mL; Sigma), and Brefeldin A (10 mg/mL; Sigma), and surface stained as described above. Intracellular staining for IL-21-producing Tfh cells was performed as described [58–60]. Briefly, cells were fixed in 2% paraformaldehyde and stored in staining buffer overnight at 4°C, permeabilized in Perm/Wash buffer (BD Pharmingen), and incubated in Perm/Wash with recombinant mouse IL-21 receptor/ human Fc chimera (5mg/mL; R&D Systems). Cells were then stained with R-Phycoerythrin-conjugated AffiniPure F(ab’)_2_ Fragment Goat Anti-Human IgG (Jackson ImmunoResearch). For the study of cytokines co-expressed with IL-21, cells were further stained with PE/Cy7 anti-IFN-γ, FITC anti-IL-10, alexa fluor 647 anti-IL-4 (all from Biolegend) or FITC anti-IFN-γ (eBioscience). Intranuclear staining of Bcl-6 was performed using PE anti-Bcl-6 (eBioscience) and the Foxp3/Transcription Factor Staining Buffer Set (eBioscience), following manufacturer’s manual. Cells were acquired on CyAn ADP (Beckman Coulter), BD FACSVerse, BD LSRII or BD LSRFortessa X-20 (BD Biosciences) flow cytometers. Dead cells were excluded by staining with LIVE/DEAD Fixable Aqua or Blue stain (Invitrogen). Singlets were selected based on FCS-A vs FCS-W and further based on SSC-A vs SSC-W. “Fluorescence minus one” (FMO) controls, in combination with isotype controls, were used to set the thresholds for positive/negative events. Analysis was performed with FlowJo software version 9.6 or higher (Tree Star).

### ELISPOT assays

ELISPOTs to measure *ex-vivo* frequencies of *P*. *chabaudi*-specific ASC in BM and MBC in spleen were performed as previously described [61]. Cells were incubated for 5h on 96-wells Multiscreen-HA filter plates (Millipore) coated with the C-terminal 21kDa part of *P*. *chabaudi* merozoite surface protein 1 (MSP1_21_)_,_ prepared as described [62], to measure the frequency of MSP1_21_-specific ASC, or coated with affinity-purified goat anti-mouse IgG (Sigma), to measure the frequency of total IgG ASC. Spots were enumerated with an Immunospot analyzer (CTL, Germany).

For MBC ELISPOTs, spleen cells were obtained by mashing the spleens through a 70 μm filter strainer, and rbc were eliminated by treatment with lysing buffer (Sigma). Cells were polyclonally activated by incubation for 6 days in flat-bottomed 96-well plates in the presence of irradiated feeder splenocytes, LPS, and supernatant from concanavalin A-stimulated C57BL/6 spleen cells. Four-fold dilutions of splenocytes were tested in replicates of 22 wells each. Cells were then transferred to antigen-coated 96-well Multiscreen-HA filter plates (Millipore) for ASC ELISPOT performed as described above. Precursor frequencies were calculated with ELDA software [63].

### ELISA

Serum samples were obtained periodically after *P*. *chabaudi* infection by bleeding the mice from the tail vein. MSP1_21_ and whole parasite lysate were generated and used in ELISAs to measure specific IgM, IgG and IgG subclasses, as previously described [64].

### Statistical analysis

Statistical analysis was performed using Mann Whitney U test or Kruskal-Wallis test on Prism software version 6 (GraphPad). P<0.05 was accepted as a statistically significant difference.

### Accession numbers

- *Ighm* Ig mu chain C region [*Mus musculus*]; Gene ID: 102641210; Uniprot ID: P01872
- *Tcra* T-cell receptor alpha chain C region [*Mus musculus*]; Gene ID: 21473; Uniprot ID: P01849
- *Rag2* V(D)J recombination-activating protein 2 [*Mus musculus*]; Gene ID: 19374; UniProt ID: P21784
- *Il21* Interleukin-21 [*Mus musculus*]; Gene ID: 60505; Uniprot ID: Q9ES17
- *Il21r* Interleukin-21 receptor [*Mus musculus*]; Gene ID: 60505; Uniprot ID: Q9JHX3
- *Bcl6* B cell leukemia/lymphoma 6 [Mus musculus]; Gene ID: 12053; Uniprot ID: P41183
- *CD16* Low affinity immunoglobulin gamma Fc region receptor III [*Mus musculus*]; Gene ID: 14131; Uniprot ID: P08508
- *CD32* Low affinity immunoglobulin gamma Fc region receptor II [*Mus musculus*]; Gene ID: 14130; Uniprot ID: P08101
- *CXCR5* chemokine (C-X-C motif) receptor 5 [*Mus musculus*]; Gene ID: 12145; UniProt ID: Q04683
- *IFNg* Interferon gamma [*Mus musculus*]; Gene ID: 15978; Uniprot ID: P01580
- *Il10* Interleukin-10 [*Mus musculus*]; Gene ID: 16153; Uniprot ID: P18893
- *TLR7* Toll like receptor 7 [*Mus musculus*]; Gene ID: 170743; Uniprot ID:Q548J0
- *Il4* Interleukin-4 [*Mus musculus*]; Gene ID: 16189; Uniprot ID: P07750
- *PD1* Programmed cell death 1 [*Mus musculus*]; Gene ID: 18566; Uniprot ID: Q02242
- *CD38* antigen [*Mus musculus*]; Gene ID: 12494; Uniprot ID: P56528
- *CD19* antigen [*Mus musculus*]; Gene ID: 12478; Uniprot ID: P25918
- *B220* protein tyrosine phosphatase, receptor type, C [*Mus musculus*]; Gene ID: 19264; Uniprot ID: P06800
- *Ter119* lymphocyte antigen 76 [*Mus musculus*]; Gene ID: 104231

## Supporting Information

S1 Fig *P*. *yoelii* infection in mice deficient in IL-21 signaling. Course of primary (A) and secondary (B) *P*. *yoelii 17X(NL)* infection in WT C57BL/6 (black circles), *Il21* ^*-/-*^ (open circles) and *Il21r* ^*-/-*^ (open squares). Statistical significance was obtained using Mann Whitney U test (*, P<0.05; **, P<0.01).
(TIF)

S2 Fig Cellular composition of the spleen during chronic *P*. *chabaudi* infection. (A) Numbers of different cell populations in the spleen of *Il21r* ^*-/-*^ and WT C57BL/6 mice at day 32 post-infection. (B) Flow cytometry gating strategy applied to identify the different cell populations. Statistical significance was obtained using Mann Whitney U test (*, P<0.05).
(TIF)

S3 Fig Gating strategy applied to identify Tfh cells in the spleen by flow cytometry. (A) Surface staining. (B) Intranuclear staining of Bcl-6.
(TIF)

S4 Fig Gating strategy applied to verify normal reconstitution in blood from mixed BM marrow chimeric mice by flow cytometry. Peripheral blood was obtained from all mixed BM chimeric mice used for experiments and analyzed by flow cytometry before infection. (A) Individual examples showing the gating strategy. (B) Frequencies of CD19^+^ B cells. (C) Frequencies of CD3^+^CD4^+^ T cells. (D) Frequencies of CD3^+^CD8^+^ T cells. No significant differences between the experimental groups and their corresponding control groups were obtained using Mann Whitney U test (P≤0.05).
(TIF)

S1 Table Combination of BM cells obtained from different donors used to reconstitute *Rag2* ^*-/-*^ mice and generate the mixed BM chimeric groups used to study the deficiency of IL-21 and IL-21R restricted to T or B cells during *P*. *chabaudi* infection. (DOCX)
