# Supplementary figures and images for: Disruption of IL-21 Signaling Affects T Cell-B Cell Interactions and Abrogates Protective Humoral Immunity to Malaria
Source: PLoS Pathog. 2015 Mar 12;11(3):e1004715. doi: 10.1371/journal.ppat.1004715 (PMC4370355; doi:10.1371/journal.ppat.1004715)

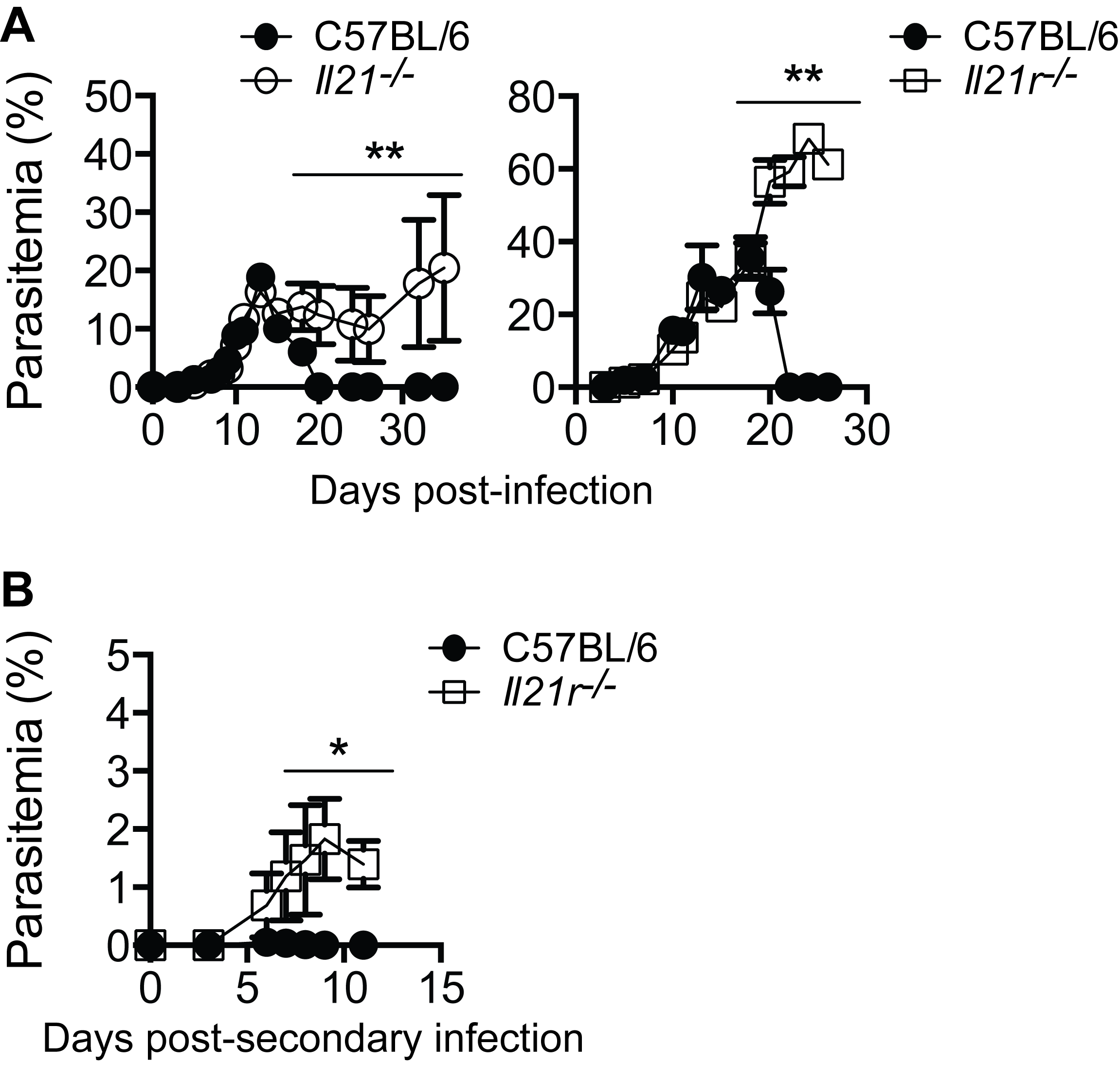

Supplement: S1 Fig — Course of primary (A) and secondary (B) P. yoelii 17X(NL) infection in WT C57BL/6 (black circles), Il21 -/- (open circles) and Il21r -/- (open squares). Statistical significance was obtained using Mann Whitney U test (*, P<0.05; **, P<0.01). (TIF) [file ppat.1004715.s001.tif]

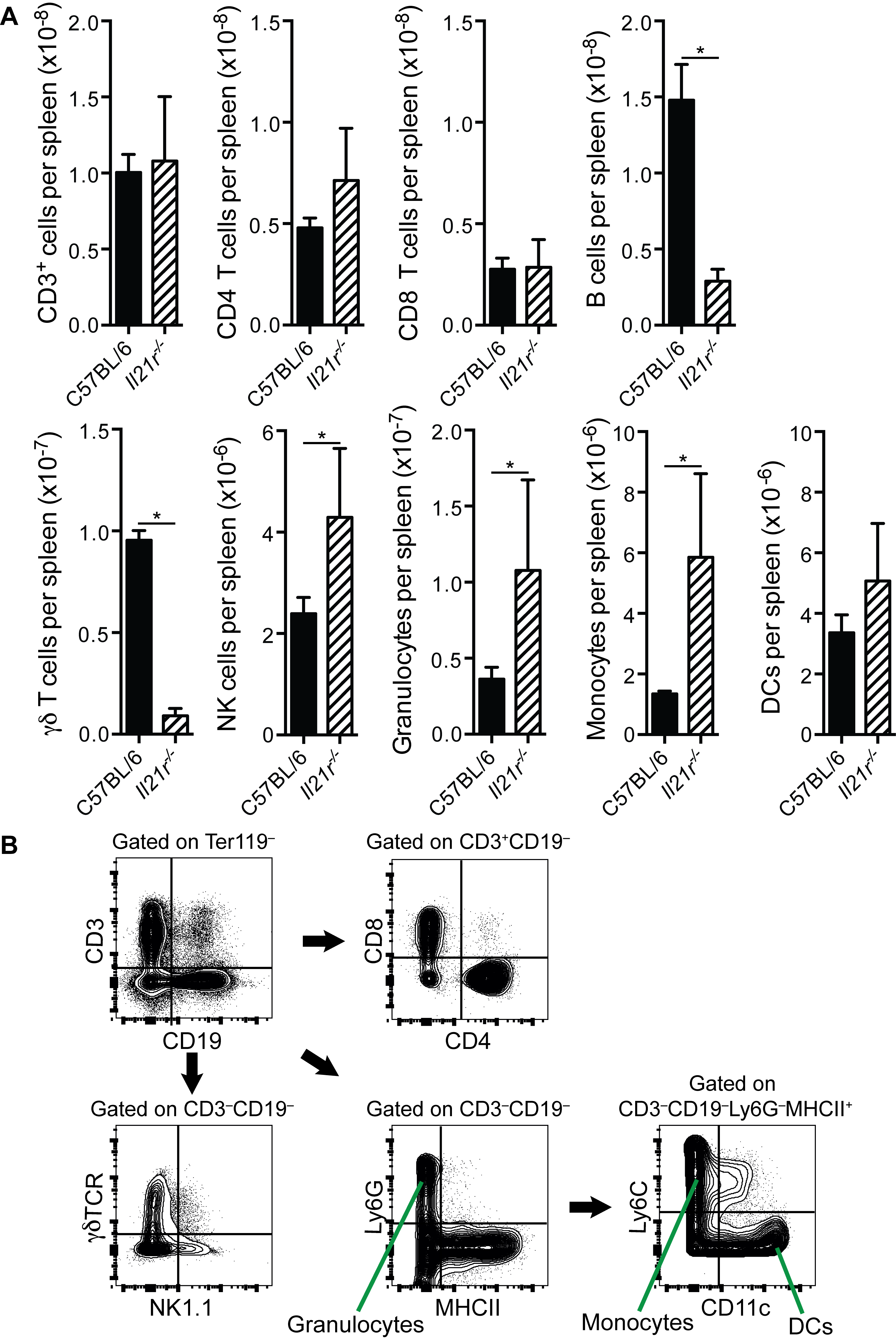

Supplement: S2 Fig — (A) Numbers of different cell populations in the spleen of Il21r -/- and WT C57BL/6 mice at day 32 post-infection. (B) Flow cytometry gating strategy applied to identify the different cell populations. Statistical significance was obtained using Mann Whitney U test (*, P<0.05). (TIF) [file ppat.1004715.s002.tif]

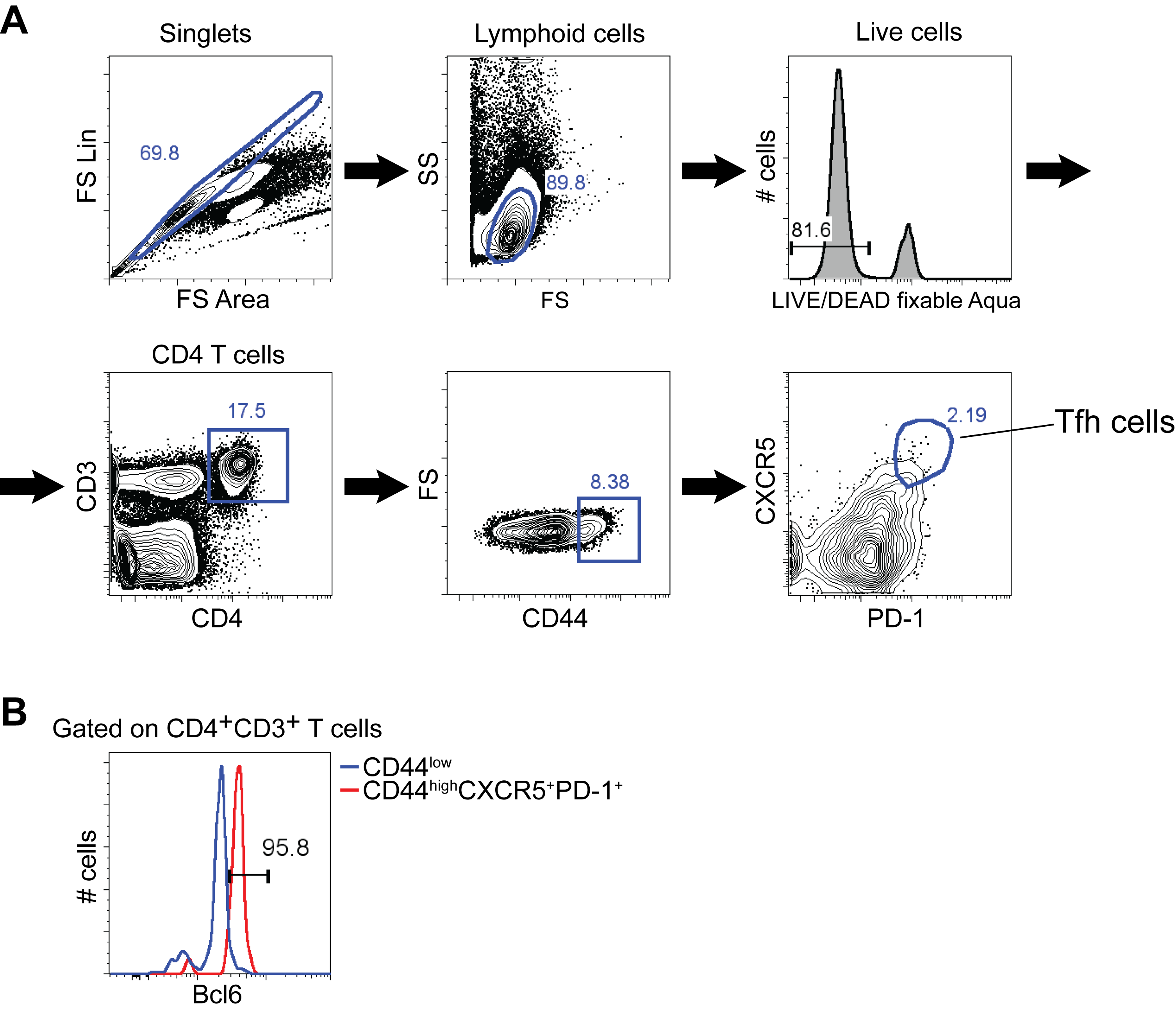

Supplement: S3 Fig — (A) Surface staining. (B) Intranuclear staining of Bcl-6. (TIF) [file ppat.1004715.s003.tif]

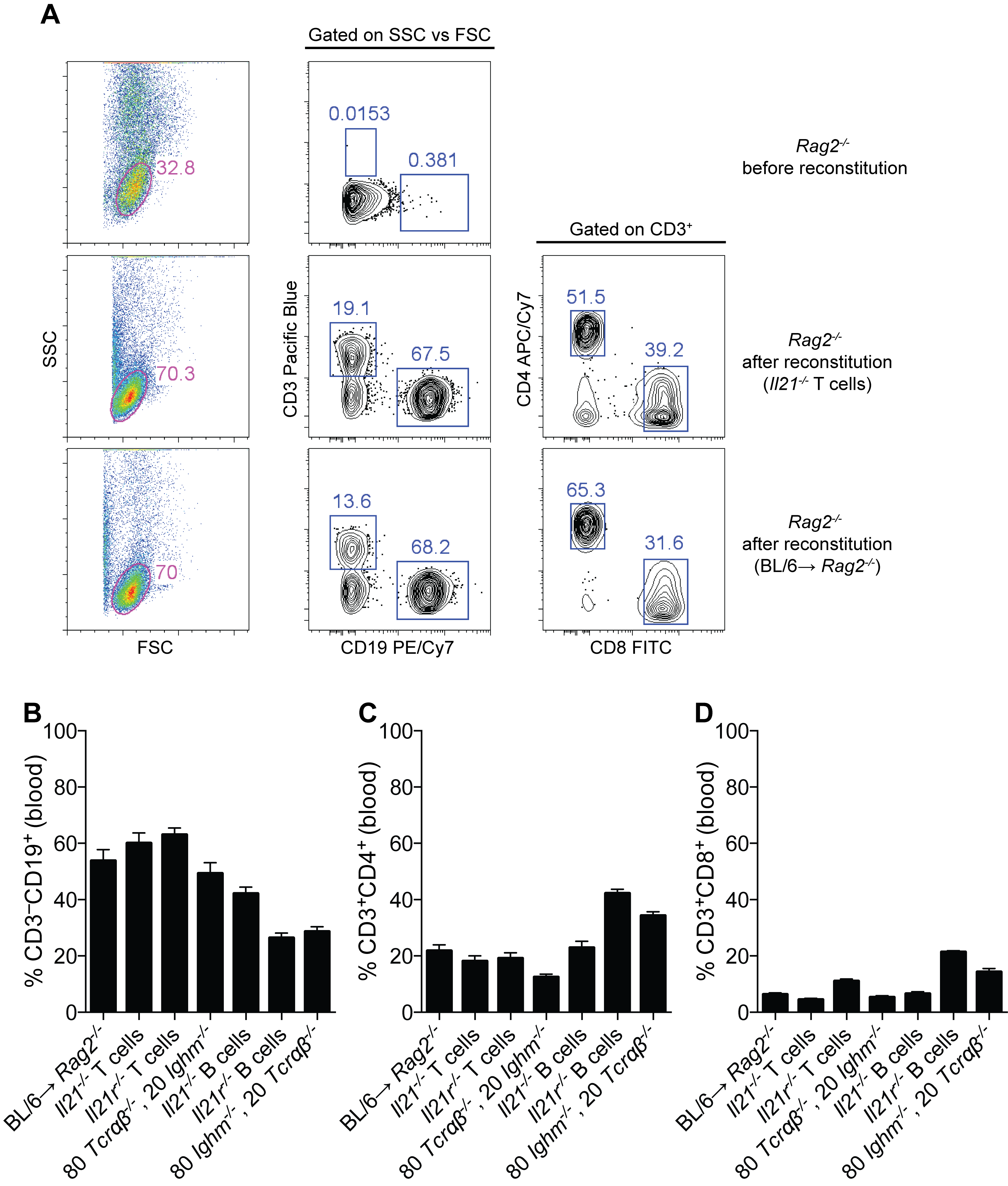

Supplement: S4 Fig — Peripheral blood was obtained from all mixed BM chimeric mice used for experiments and analyzed by flow cytometry before infection. (A) Individual examples showing the gating strategy. (B) Frequencies of CD19+ B cells. (C) Frequencies of CD3+CD4+ T cells. (D) Frequencies of CD3+CD8+ T cells. No significant differences between the experimental groups and their corresponding control groups were obtained using Mann Whitney U test (P≤0.05). (TIF) [file ppat.1004715.s004.tif]
