# Supplementary material for: Disruption of IL-21 Signaling Affects T Cell-B Cell Interactions and Abrogates Protective Humoral Immunity to Malaria
Source: PLoS Pathog. 2015 Mar 12;11(3):e1004715. doi: 10.1371/journal.ppat.1004715 (PMC4370355; doi:10.1371/journal.ppat.1004715)
Supplement: S1 Table — (DOCX) [file ppat.1004715.s005.docx]

**Table S1.**  Combination of BM cells obtained from different donors used to reconstitute *Rag2^-/-^* mice and generate the mixed BM chimeric groups used to study the deficiency of IL-21 and IL-21R restricted to T or B cells during *P. chabaudi* infection.

| Group | Donor 1^a^ | | | Donor 2 | | | Phenotype |
| --- | --- | --- | --- | --- | --- | --- | --- |
|  | Strain | N° cells (x 10^-6^) | % cells | Strain | N° cells (x 10^-6^) | % cells |  |
| BL/6*→ Rag2^-/-^* | C57BL/6 | 4 | 100 | – | – | – | Control WT |
| 80 *Tcra^-/-^*, 20 *Ighm* | *Tcra^-/-^* | 3.2 | 80 | *Ighm* | 0.8 | 20 | Control WT |
| 80 *Ighm*, 20 *Tcra^-/-^* | *Ighm* | 3.2 | 80 | *Tcra^-/-^* | 0.8 | 20 | Control WT |
| *Il21^-/-^* T cells | *Tcra^-/-^* | 3.2 | 80 | *Ighm*, *Il21^-/-^* | 0.8 | 20 | T cells deficient in IL-21 |
| *Il21r^-/-^* T cells | *Tcra^-/-^* | 3.2 | 80 | *Ighm*, *Il21r^-/-^* | 0.8 | 20 | T cells deficient in IL-21R |
| *Il21^-/-^* B cells | *Ighm* | 3.2 | 80 | *Tcra^-/-^*, *Il21^-/-^* | 0.8 | 20 | B cells deficient in IL-21 |
| *Il21r^-/-^* B cells | *Ighm* | 3.2 | 80 | *Tcra^-/-^*, *Il21r^-/-^* | 0.8 | 20 | B cells deficient in IL-21R |

^a^ BM cells were obtained from different donors as described in Materials and Methods and combined in different proportions to obtain a mixture containing 4x10^6^ total cells. This mixture was injected i.v. into sub-lethally irradiated *Rag2^-/-^* mice.
